# Research on the Heterogeneity of Carbon Emissions under the Government’s Promotion of Urban Agglomeration Development: Empirical Evidence from County-to-District Reforms

**DOI:** 10.3390/ijerph19127540

**Published:** 2022-06-20

**Authors:** Jing Jin, Duozhang Chen

**Affiliations:** School of Management, Zhejiang University of Technology, Hangzhou 310023, China; jinjing772019@163.com

**Keywords:** county-to-district reform, per capita carbon emissions, county, center-periphery, Yangtze River Delta city cluster, regional integration

## Abstract

County-to-district reform (CTDR) is an important policy path for the government to promote the cultivation and construction of urban agglomerations, and exploring its “carbon emission” effect is of great significance for the high-quality development of urban agglomerations and the realization of the “dual carbon” goal. Based on the panel data of 120 counties in the Yangtze River Delta urban agglomeration from 2000–2017, this paper empirically tests the effect of county-to-district reforms on per capita carbon emissions in the counties of the central and peripheral cities of the Yangtze River Delta urban agglomeration under the Kutznets curve (EKC) hypothesis and the integrated difference-in-difference (DID) model and STIRPAT model. The results show that: (1) The carbon emission effect of county-to-district reforms have significant regional heterogeneity. The reforms of the central city of the urban agglomeration significantly reduced the per capita carbon emission of the county by 4.27%, whereas the reforms of the periphery cities of the urban agglomeration significantly increased per capita carbon emission by 6.56%. (2) The impact of county-to-district reforms on county per capita carbon emissions began to appear in the fourth year of reform. (3) Mechanism analysis showed that county-to-district reforms promoted central cities population agglomeration and reduction of carbon emission intensity can help reduce the per capita carbon emission level in counties, whereas peripheral cities have a dual carbon-increasing effect of decreasing population density and increasing carbon emission intensity. Therefore, the approval of county-to-district reforms should be strictly controlled, and the reform of non-central cities would be especially prudent, so as to reduce the negative effect of reform on the high-quality development of cities.

## 1. Introduction

Over the past 40 years of reform and opening up, China has experienced a dual pattern of rapid economic growth and high carbon emissions. The greenhouse effect of excessive carbon emissions is already contributing to global warming and sea level rise. In September 2020, President Xi Jinping proposed China’s “dual carbon strategy” at the 75th United Nations General Assembly; that is, through technological innovation and energy conservation and emission reduction, China will achieve “carbon peak” in 2030 and “carbon neutrality” in 2060. As the world’s largest developing country, China’s carbon emissions have been hovering at a high level, with the total fluctuating around 9.3 billion tons [1]. With the people’s desire for a better life and better health, green and low carbon have become the new path of China’s development. In the production sector, in 2019, Zhejiang, the birthplace of the “Two Mountains Theory”, began to implement a comprehensive reform of industrial land (“standard land”), which emphasizes the efficient output and intensive use of industrial land but also limits high energy consumption and high emissions. In the consumer sector, the nationwide promotion of the “new energy vehicle consumption” policy can greatly reduce harmful emissions of carbon dioxide and other gases. China’s urbanization rate has increased from 17.9% (1978) to 63.89% (data from the 7th census in 2020), with an average annual growth rate of 1.09%. Although China is in the middle stage of urbanization (30–70%) [2], rapid urbanization is the result of the transition from primary to secondary and tertiary industries, which attracts a large number of rural laborers to the cities. The rapid growth and expansion of energy, transportation, construction and industry promote urban carbon emissions [3,4]. Urban agglomerations have become the growth poles and important spatial forms of China’s economy. The policy reform of the promising government has greatly promoted the development and construction of urban agglomerations, including spatial regional planning, development zone construction, priority development of provincial capital cities and administrative divisions. Among them, the reform of administrative divisions has significantly promoted the development of urban agglomerations [5]. 

“Carbon emissions” has become one of the key concerns of environmental scholars, geographers and economists in the past decade. First, scholars have studied the spatial and temporal evolution patterns and influencing factors of carbon emissions in different regions from a geographic perspective [6,7]. Second, scholars mainly discuss the relationship between economic development factors and carbon emissions based on urbanization [8], economic development [9], investment [10] and transportation [11]. Third, research methods mainly include the LMDI index decomposition method [12], the geographically weighted regression model [8], the spatial panel data model [13] and other methods to explore the impact of different variables on carbon emissions.

Government actions and policies promote economic growth, but environmental effects still deserve attention. Guo et al. (2022) regarded the promulgation of the “Regional Planning for the Yangtze River Delta Region” in 2010 as a quasi-natural experiment and empirically assessed that the regional integration of the Yangtze River Delta significantly reduced the carbon emissions of cities in the Yangtze River Delta [14]. Yu et al. (2020) found that central and local governments choose different paths in promoting economic low-carbon development from an industrial planning perspective [15]. World cities such as New York, London and Paris play a role in carbon reduction through spatial planning interventions in areas such as transportation, buildings and land resources [16]. Spatial structure optimization is an important way to achieve low-carbon cities [17]. The economic agglomeration effect of development zones and industrial parks is beneficial to reduce carbon emission intensity [18]. Regional market integration significantly enhances regional carbon benefits [19], whereas market segmentation increases regional carbon emissions [20,21]. The above studies mainly focus on cities or provinces, but few studies take counties as the research unit, which are important economic and administrative units in China. Few scholars explore the relationship with carbon emissions from the perspective of administrative division policies such as county-to-district (municipal district) reform. What impact will reform have on the carbon emissions of urban agglomerations? Will there be industrial differences between the central and peripheral cities within the urban agglomeration?

The study of the impact of county-to-district reforms on carbon emissions can focus on two aspects:

On the one hand, county-to-district reforms expand urban space and promote urbanization. Scholars have conducted numerous studies on the relationship between urbanization and carbon emissions but have not reached a consistent conclusion. Some scholars consider a linear relationship between urbanization and carbon emissions and find that urbanization rate significantly contributes to carbon emissions through the Yangtze River Delta, Pearl River Delta city cluster and inter-provincial panel data in China [22,23,24] and that the effect of urbanization rate on carbon emissions is regionally heterogeneous [25]. Other scholars have found a positive or negative non-linear relationship between urbanization rate and carbon emissions [26]. 

On the other hand, county-to-district reforms have been extensively empirically confirmed to have boosted regional economic growth [27,28]. The relationship between economic development and carbon emissions also shows various results. Zhao et al. (2013) found that economic growth promotes carbon emissions both in the short and long term [29]. Some scholars have found that GDP and environmental pollution satisfy the Kuznets curve (EKC). At the beginning of economic development, carbon emissions curve with GDP growth, and when a certain threshold is reached, economies of scale and technological advances help mitigate carbon emissions [30].

In the face of divergent conclusions, county-to-district reform provides a new methodological perspective to test the impact of urbanization and economic development on carbon emissions. Therefore, based on the county panel data of the Yangtze River Delta urban agglomeration from 2000 to 2017, this paper uses the difference in difference method to test the impact of county-to-district reforms on county carbon emissions, focusing on the heterogeneity between urban agglomeration centers and peripheral cities. The possible marginal contributions of this paper are as follows: (1) to provide a new empirical perspective and methodology for studying the impact of regional integration on carbon emissions through the quasi-natural experiment of county abolition reform; (2) to complement the policy evaluation effects of county-to-district reforms; (3) to provide relevant policy empirical support for China’s “carbon emission reduction” and “dual carbon strategy”.

## 2. Policy Background and Study Area

In China, counties and municipal districts are both county-level administrative units. Municipal districts are dominated by urban population and non-agricultural industries. The financial and urban management rights of municipal districts are under the unified management of prefecture-level cities, whereas counties are relatively independent. Municipal districts and counties have the characteristics of centralization and decentralization, respectively. The Ministry of Civil Affairs of China formulated the Standards for the Establishment of Municipal Districts, which set standards for the city’s industrial structure, population size, fiscal revenue, economic aggregate and other indicators. County-to-district reform is conducive to increasing urban space and plays an especially important role in enhancing the comprehensive development level of central cities and promoting the development of urban agglomerations.

In the 70 years since the founding of the PRC, the main administrative means of reorganizing China’s administrative divisions have included the abolition of counties and the establishment of districts and cities. Counties-to-cities reforms had been suspended due to inefficiency (1997~2016), and the list of cities approved by the Ministry of Civil Affairs for removal of counties to establish cities in the past three years is basically dominated by small and medium-sized cities in Central and Western China, whereas county-to-district reform has become an important means to promote the development process of urbanization in China. China’s county-to-district reform began in the 1980s and has developed rapidly. As of December 2018, China has seen municipalities directly under the central government (Beijing, Shanghai, Tianjin) and prefecture-level cities (Wuhan, Nanjing, Guangzhou, Shenzhen, Xiamen, Zhuhai, Zhongshan, Dongguan, Foshan) presenting urban characteristics without counties. China has seen 181 reforms of county abolition (1999~2018), including 104 reformed cities, which are widely distributed in major cities in East, Central and West China. Eastern cities and provincial capitals are hotspots for reform. Since factors such as economy, population and land area are important indicators for the State Council to approve whether a city can pass the reform, the county-to-district reform is non-random in nature. As shown inFigure 1, in terms of time distribution, 2000–2004 and after 2010 are the boom periods of county-to-district reforms. In the future, China has a strong preference for local governments to county-to-district reforms in the context of the key development of urban clusters and metropolitan areas. County-to-district reforms effectively break the border effect between counties and cities and the dual structure of urban–rural division and promote the integrated construction and development of cities. In March 2022, China’s State Council issued “the Opinions on Accelerating the Construction of a Unified National Market”, and county-to-district reform, as an effective policy tool for the government to promote the construction of regional integration, has provided policy support to facilitate the flow of resource factors (land, human resources, capital, information, etc.).

The Yangtze River Delta city cluster is used as the object of this paper for the following reasons:

First, the Yangtze River Delta city cluster, as the most economically developed city cluster in China, covers 26 cities, including Shanghai, Nanjing, Hangzhou, Hefei, Ningbo, Suzhou, Wuxi, etc. The Yangtze River Delta city cluster only accounts for 2.3% of the national land area but gathers 225 million people and contributes 25% of the national GDP. Reforms occurred frequently, with 30 in total, accounting for about 17% of the total number of reforms nationwide.

**Figure 2:**
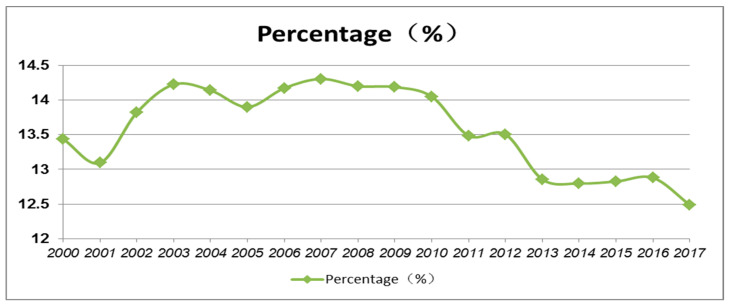
Carbon Emissions of Yangtze River Delta Urban Agglomerations as a Percentage of China’s Carbon Emissions, 2000–2017. Data source: Calculated based on the county’s annual carbon emissions.

Second, the urbanization rate of the Yangtze River Delta urban agglomeration is as high as 69% (2017), and cities such as Shanghai, Hangzhou, Nanjing and Ningbo have carried out a large number of county abolitions due to spatial development constraints, which has promoted regional economic growth and urban agglomeration development; but from 2000–2017, the carbon emission ratio of the Yangtze River Delta urban agglomeration was as high as 12~14.5% of the country, and after 2008, the carbon emission ratio of the Yangtze River Delta urban agglomeration declined year by year after 2008 (Figure 2). 

Third, there are significant differences in carbon emission levels among different provinces and cities within urban agglomerations, and the regional heterogeneity of reform can be verified.

## 3. Mechanism Analysis

There is a paucity of literature directly examining the relationship between county-to-district reforms and carbon emissions. Numerous empirical studies have demonstrated that county-to-district reform promotes regional economic growth. Panayotou (1993) [31] first introduced the concept of the environmental Kuznets curve, in which economic growth and environmental pollution are no longer in a mutually “exclusive” relationship. Economic growth degrades the environment through scale effects on the one hand and optimizes it through technological and structural effects on the other. In the stage of rapid economic development, the scale effect exceeds the technology and structure effects, and the overall environment deteriorates, and when the economy reaches a certain threshold stage, the technology and structure effects gradually overtake the scale effect [32]. Therefore, county-to-district reforms may affect per capita carbon emissions through agglomeration, technology and structural effects.

First, county-to-district reform promotes industrial agglomeration [33]. The negative externality of agglomeration is manifested as a shift from an agricultural- to an industry-led urban economy and is accompanied by low to high carbon emissions. “People follow the industry”. County-to-district reform promotes population clustering and thus increases the level of population density [34]. The positive externalities of agglomeration exhibit economies of scale and significantly reduce the level of per capita carbon emissions in the county. In the left phase of the inverted U-shaped curve of economic development and environmental pollution, the scale effect clearly promotes per capita carbon emissions.

Second, county-to-district reform has promoted business innovation and improved the business environment in counties [35]. Environmental regulatory pressures drive companies to change technology, and innovations in technology can help improve energy efficiency and thus reduce carbon emissions per capita. China is in a critical period of socialist economic development, and to eradicate the extreme negative thinking of “no development, no pollution”, improving energy efficiency has become the most important path to balanced development [36]. Both in central and peripheral cities, improving energy use efficiency contributes to lower carbon emissions per capita, but technological upgrading is stronger in central cities than in peripheral cities. County-to-district reform promotes government public spending [37], and the positive externalities of public spending on the environment contribute to lower levels of carbon emissions per capita. Government public spending is higher in central cities than in peripheral cities, and thus the carbon reduction effect is stronger in central cities. Since the economic output value of peripheral cities dominated by secondary industries is lower than that of central cities dominated by tertiary industries, and at the same time the secondary industries release more carbon emissions, the carbon emission intensity of peripheral cities is higher than that of central cities. Central cities have a higher concentration effect of talent and technological innovation and are more likely to improve energy use efficiency. Therefore, the carbon reduction effect is stronger in central cities.

Third, the county-to-district reform changed the industrial structure of the region [38]. County to district is a span from a primary sector rural economy to a secondary- and tertiary-sector-oriented urban economy. For the central city, the proportion of tertiary industry is higher than that of secondary industry, whereas the peripheral cities will take up part of the spillover of secondary industry from the central city. The county already had a certain industrial base before the annexation, and stricter environmental regulation policies may be imposed under the municipal jurisdiction; the secondary industry may decrease, and the tertiary industry may increase in leaps and bounds after the annexation. Therefore, the county-to-district reforms may optimize the industrial structure of counties and thus reduce carbon emissions per capita.

## 4. Description of Research Methods and Variables

### 4.1. Difference-in-Difference Model

In this paper, we choose a DID model for empirical testing [39], and the DID method is a common method for policy effect evaluation. In this paper, we set the econometric equation of the impact of county abolition on carbon emissions (CE) as:

(1) $$CE_{it}=\alpha +\beta_{1}Reform_{i}\times After_{t}+\lambda \underset{n}{\sum }Controls_{it}+\gamma_{t}+\mu_{i}+\varepsilon_{it}$$

In Equation (1), the subscript *i* represents the county, *t* is the time and *CE* is the carbon emission index. The sample of this period occurred during the abolition of the county reform. *Reform* takes the value of 1; otherwise, it takes 0. If the time of county abolition reform is *t*, *After* takes 1 after year *t*; otherwise, it takes 0. *Controls* are the control variables that affect the change of carbon emission level with county and time, and *ε* is the residual. Due to the inconsistency of reform time in different places, the study adopted a multi-period double-difference method to include all counties without county abolition reform as the control group and counties with county abolition reform as the experimental group. When the interaction term *Reform* × *After* is 1, a county *i* has specifically carried out the reform of county-to-district in a certain year *t*. Otherwise, the DID interaction term of this county is equal to 0. In this paper, we control for both time fixed effects $\gamma_{t}$ and county fixed effects $\mu_{i}$ to exclude the effects of time factors and individual county factors.

### 4.2. STIRPAT Model

The STIRPAT model is derived from the IPAT benchmark model. The impact of the environment (I) comes from population (P), affluence (A), and technology (T) [40]. Based on the extended STIRPAT model [41], the key factors affecting carbon emissions (**CE**) are:

(1) Population factor. Population influences carbon emissions through its own production (supply) and consumption (demand) behaviors [42], and population density (**PD**) better reflects the agglomeration effect of population than the absolute indicator population size [43];
(2) Economic level, economy and carbon emission show diversified grouping. In this paper, we choose regional GDP, the secondary term of regional GDP, and the tertiary term of regional GDP to test whether the relationship between economic development and carbon emissions is consistent with the environmental Kuznets (EKC) hypothesis. In addition, local government public expenditure (**GPE**) is supplemented to measure the level of local economic development;
(3) Industrial structure. Carbon emissions mainly come from secondary industry, and the value added by secondary industry (**SI**) is selected to explore the impact of the secondary industry on carbon emissions [44];
(4) Technology level. This paper uses carbon emission intensity (**CEI**) to characterize the impact of technology level on carbon emissions [45,46].

All variables are taken logarithmically, and their expressions are shown in Equation (2).

ln(*CE*)_*it*_ = *α*_0_ + *β*_2_ ln*PD*_*it*_ + *β*_3_ ln*GDP*_*it*_ + *β*_4_(ln*GDP*_*it*_)^2^ +*β*_5_(ln*GDP*_*it*_)^3^ + *β*_6_ ln*GPE*_*it*_ + *β*_7_ ln*CEI*_*it*_ + *β*_8_ ln *SI*_*it*_ + *ε*_*it*_ (2)

The different coefficients and significance of the above equation can determine the factors affecting carbon emissions. Table 1 mainly shows the relationship between GDP and carbon emissions for different signs, taking values of *β*_3_, *β*_4_ and *β*_5_.

Equations (1) and (2) were combined and a panel data regression analysis was performed according to Equation (3).

(3) $${\ln(CE)}_{it}=\alpha_{0}+\beta_{1}Reform_{i}*After_{t}+\beta_{2}\;{\ln PD}_{it}+\beta_{3}\;{\ln GDP}_{it}+\beta_{4}({\ln GDP}_{it}{)}^{2}+\beta_{5}({\ln GDP}_{it}{)}^{3}+\beta_{6}\;{\ln GPE}_{it}+\beta_{7}\;{\ln CEI}_{it}+\beta_{8}\;\ln\;{SI}_{it}+\gamma_{t}+\mu_{i}+\varepsilon_{it}\;$$

### 4.3. Variable Description and Descriptive Statistics

Explained variables, per capita carbon emissions (**PCE**). The balanced development between regions pays more attention to the per capita level; the population agglomeration increases the total carbon emissions, but the per capita carbon emissions can more accurately measure the evolution of carbon emission level between regions. The carbon emissions per capita in county areas are taken as logarithms, and the carbon emissions data of county units in CEADS China Carbon Emission Database are used in this paper. The current study of carbon emission data mainly comes from energy consumption data. Measuring carbon emission data from provincial and prefecture-level cities in China, without measuring carbon emission data from county level, has generated great research limitations. The research unit of this paper is county-based, which can more accurately reflect the “net” impact of county-to-district reforms on per capita carbon emissions. The nighttime light data used in previous studies of carbon emissions are biased in terms of measurement accuracy. The carbon emissions data provided in this database were estimated for Chinese counties using the particle swarm optimization-back propagation (PSO-BP) algorithm to unify the scale of DMSP/OLS and NPP/VIIRS satellite images.

Explanatory variables, reform variables for the county-to-district reforms. Source from the official website of the Ministry of Civil Affairs of the People’s Republic of China (http://www.mca.gov.cn) (accessed on 1 January 2022). We manually compiled the administrative division adjustment documents issued by the Ministry of Civil Affairs of the State Council from 1999 to 2017 and the Handbook of Administrative Divisions of China (1999~2017). Due to the low frequency of county abolition occurring in the region before 1999, the study was not considered. At the end of 1999, there were 120 county-level (county and county-level city) administrative units in the Yangtze River Delta urban agglomeration, excluding district-level administrative units, that had completed their changes. From 2000–2017, a total of 30 county-level administrative units completed the county-to-district reforms, including Qingpu District in Shanghai, which completed the county-to-district reform at the end of 1999 and joined the experimental group. These 30 counties constitute the experimental group of the study, and the remaining counties (cities) are the control group. 

Controlling variables. This paper controls for the main influencing factors that affect regional carbon emissions. The data were obtained from the 2000–2017 China County Statistical Yearbook and the statistical yearbooks of cities in Shanghai, Zhejiang, Jiangsu, and Anhui Provinces, China.

The results of descriptive statistics for each variable are shown in Table 2. To eliminate the effects of inflation, GDP, secondary sector value added and government public expenditure are discounted to constant year 2000 by the GDP deflator.

## 5. Empirical Analysis

### 5.1. Benchmark Effect

Randomness tests were performed by logistic regression, and parallel trend tests were performed to satisfy the conditions for the use of the DID model. The overall impact effect of reform on county carbon emissions is tested by Equation (3). Columns 3 and 4 in Table 3 add all control variables, and columns 2 and 4 control for fixed effects. The results show that with or without the inclusion of control variables, the county abolition reform significantly increases the per capita carbon emission level of the reformed counties, and column 4 illustrates that the county abolition reform significantly increases the per capita carbon emission level of the counties by 1.63%. Population density is inversely proportional to per capita carbon emissions, indicating that the agglomeration effect of population helps to curb the increase of per capita carbon emissions. The value added and carbon intensity of secondary industries significantly increase the level of carbon emissions per capita. Therefore, optimizing industrial structure, innovating technology and improving energy use efficiency are conducive to reducing the level of carbon emissions per capita. While the coefficient of government public expenditure is significantly negative, the increase of government public expenditure presents a positive economic externality effect and improves the regional per capita carbon emission level. Economic development and per capita carbon emissions show an inverted N-shaped relationship, as judged by the sign of GDP. The inverted N-shaped curve shows two inflection points, compared with the inverted U-shaped curve of economic development and environmental pollution, which has only one inflection point. Economic development and carbon emissions show a relationship of first decrease, then increase and then decrease. At the early stage of economic development, county production activities are influenced by the agglomeration effect and the economy-of-scale effect, which can effectively level off the increase of per capita carbon emissions. However, under the excessive use of fossil materials and the government’s low-standard environmental policies, carbon emissions began to rise and the first inflection point occurred. As overcapacity is eliminated from the market and new industries sprout and develop, coupled with strict government policies on environmental regulation, carbon emissions per capita once again decline.

### 5.2. Heterogeneity Test Based on “Center-Periphery”

The Yangtze River Delta region has a developed economy, but there are large differences between the central cities and peripheral cities within the city cluster. The cities are divided into two groups of 24 counties in central cities (Shanghai, Hangzhou, Nanjing, Ningbo and Hefei) and 96 counties in non-central cities (the rest of the cities) to study the impact of county-to-district reforms on per capita carbon emissions. The calculation results show (Table 4) that the effect of county-to-district reforms from central cities on per capita carbon emissions is significantly negative with or without the inclusion of control variables. It indicates that the central cities show positive externalities of the reform. For peripheral cities, the removal of counties and districts significantly increases the level of carbon emissions per capita in the county. From the cross-term coefficients, the absolute values of the peripheral coefficients are all greater than the absolute values of the central coefficients. Therefore, this is consistent with the positive coefficients of the interaction terms in the overall benchmark regression. The control variable coefficient of value added by secondary industry in central cities no longer has a significant effect on per capita carbon emissions, probably because central cities show the dominance of tertiary industry in the industrial structure. In the relationship between GDP and carbon emission per capita, the peripheral cities show an inverted “N” curve relationship, and the central cities show an “N” curve relationship, so we can judge that when the central cities increase their GDP values and carbon emission per capita decreases, the peripheral cities show an increasing trend. The coefficients of the remaining control variables are similar to the base regression. This shows that the effect of county abolition on per capita carbon emissions is spatially heterogeneous.

### 5.3. Placebo Test

To improve the credibility and rigor of the estimation results, counterfactual tests, specifically the placebo test, were conducted in this paper. Specific approach: set the year of county abolition to 1, otherwise 0. A year ahead of schedule, set to 1, otherwise 0. Set in sequence to the 5th year before the reform. The regression results of substituting the above six variables for the variable Reform ∗ After are shown in column (1) of Table 5. All six variables were insignificant and had negative coefficients in some years. It shows that if the county-to-district reform occurs earlier, it will not have a significant impact on per capita carbon emissions.

The temporal effect of county abolition on carbon emissions was further examined. It is 1 in the first year after setting up the abolition of the county; otherwise, it is 0. It is 1 in the second year after the reform, otherwise 0. Set in sequence until the 5th year after the reform. The regression results are shown in column (2) of Table 5, where the effect of the reform starts to appear in year 4, which matches the results of Zhuang’s [39] study on economic growth.

### 5.4. Further Robustness Tests

In order to test the stability of the effect of county abolition on per capita carbon emission, this paper selects the time periods 2000–2003 and 2009–2017 to test the effect of county abolition on per capita carbon emission in the Yangtze River Delta city cluster. As shown in columns (1) and (3) of Table 6, the absolute values of the coefficients are greater than the baseline regression result of 0.0163, indicating that county-to-district reform significantly increases the level of carbon emissions per capita. Columns (2) and (4) show that county-to-district reform significantly contributes to the per capita carbon emissions of peripheral cities. The absolute values of the coefficients in columns (2) and (4) are larger than those in columns (1) and (3), indicating that the direction of the effect of central city removal on per capita carbon emissions is negative. Due to the small sample of central cities, the results are prone to bias and are not shown. The above results demonstrate the stability of the impact of county removal on per capita carbon emissions.

### 5.5. Mechanism Testing

The baseline regression shows that increasing population density significantly reduces carbon emissions per capita and increasing carbon intensity significantly increases carbon emissions per capita. The larger the value of secondary industry than tertiary industry, the lower the degree of industrial structure optimization and the greater the carbon emission intensity per capita. Among them, in terms of coefficients, carbon intensity has the greatest impact on per capita carbon emissions, so improving energy efficiency becomes the best way to reduce carbon emissions. This section examines the effects of county abolition on county population density, carbon emission intensity and the ratio of the secondary sector over the tertiary sector. As shown in Table 7, overall, the county-to-district reform reduces the carbon emission intensity and the ratio of secondary industry to tertiary industry (***SI/TI***). It indicates that the county-to-district reform has significantly improved the efficiency of energy use and promoted the upgrading of industrial structure. Sub-regionally, the county-to-district reform promotes population concentration in central cities and reduces population density in peripheral cities; reduces carbon emission intensity in central cities and increases carbon emission intensity in peripheral cities; and optimizes the upgrading of urban industrial structure but is not significant for central cities. On the one hand, central cities have lower per capita carbon emissions due to much more efficient energy use than peripheral cities. On the other hand, the positive externalities of population clustering are stronger. Therefore, county-to-district reform promotes the reduction of emissions in central cities and the increase of emissions in peripheral cities.

## 6. Conclusions and Recommendations

This paper selects the Yangtze River Delta urban agglomeration, which is economically developed, with more frequent county abolition and larger carbon emissions, as the study area, and systematically examines the “net” impact of county abolition on per capita carbon emissions with the help of a DID model and STIRPAT model, using county panel data of 120 counties (cities) from 2000 to 2017, focusing on the heterogeneity between urban agglomeration centers and peripheral cities.

The study draws the following four conclusions:

(1) County-to-district reform has contributed to an average of 1.63% of carbon emissions per capita;
(2) At the city level, the reform of cities from the center of the urban agglomeration significantly reduces carbon emissions per capita in the county by 4.27%, whereas the reform of cities from the periphery of the urban agglomeration significantly increases carbon emissions per capita by 6.56%;
(3) The impact of county abolition on per capita carbon emissions in counties starts to appear from the fourth year of the reform;
(4) The mechanism analysis shows that county-to-district reform promotes population agglomeration, industrial structure upgrading, and improved technological innovation to help reduce the per capita carbon emission level of counties. The population and technological innovation are concentrated in the central cities, whereas the opposite is true for the peripheral cities, thus creating a difference in the impact of county removal on carbon emissions in the central and peripheral cities.

The impact of both urbanization and economic development on carbon emissions is reflected in different areas and aspects of carbon emissions at different stages of development. Within the same urban agglomeration, different cities are at different stages of development. Most of the central cities have entered the era of sophisticated high iteration of innovation; a small number of central cities and some peripheral cities are at the scale capital intensive stage and most peripheral cities are still at the labor intensive stage, while different stages drive differentiated carbon emissions. Therefore, the conclusions based on the available data tests do not negate the effectiveness of the policy of county-to-district reform. The difference between the county-to-district reform on the central and peripheral cities of the urban agglomeration is also revealed by the different stages of development. At this stage, the level of integration of central cities is better than that of peripheral cities. In this paper, we find that the disintegration creates differences in population concentration levels and energy use efficiency between the central and peripheral regions, resulting in divergent carbon emission levels.

The results of this paper are an effective supplement to the evaluation of administrative reform policies on county-to-district reform, while providing theoretical support for urban cluster development and carbon emission reduction. In view of the above conclusions, it is recommended that the following actions be taken:

(1) Prohibit local governments from blindly promoting county-to-district reform, i.e., strictly control the approval of county-to-district reform. Be sure to comply with the laws of economic and social development; for example, the population scale agglomeration effect formed by county-to-district reform in central cities and the improvement of energy use efficiency can help curb carbon emissions.
(2) Promote population clustering in urban agglomerations, increase population density, and gradually reduce or even eliminate institutional constraints on labor mobility. Prevent the sprawling development of the land and use the land intensively and economically.
(3) Improve the efficiency of enterprises’ energy use through technological innovation [47,48] and especially policies that encourage the flow of innovation factors (talent, capital, technology, etc.) to peripheral cities, further promoting the integrated development of urban clusters. The agglomeration effect and policy advantages of central cities are conducive to carbon emission reduction; therefore, improve the energy utilization efficiency of all cities, and gradually restrain the increase of per capita carbon emissions in peripheral cities, thereby reducing the overall carbon emissions of urban agglomerations. In short, peripheral cities cannot become victims of central city development and economic development. On the one hand, some cities can take advantage of their own resource endowments to fully develop agriculture and tourism. On the other hand, cities that rely on industrial energy can explore ways to improve energy utilization efficiency.
(4) The government and enterprises should make full use of the objective laws of economic development—for example, maintaining the dual effect of energy saving and emission reduction in the declining phase of the inverted “N” curve of the relationship between economic development and per capita carbon emissions [49].

In addition, the transfer of high-carbon emission enterprises may be an important reason for the increase of carbon emissions in peripheral cities. Due to data limitations, the authors did not explore this factor in the mechanism test, which provides ideas for further research in the future.

## Figures and Tables

**Figure 1:**
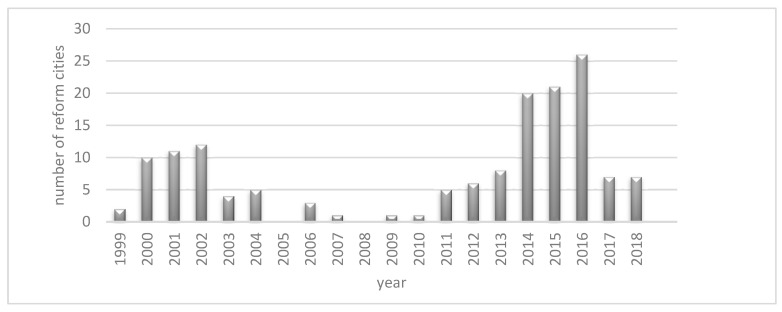
Number of county-to-district reforms in China, 1999–2018. Data source: Calculated based on all reform documents.

**Table 1:** Different relationships between GDP and carbon emissions.

Coefficients			Implication
*β*_3_	*β*_4_	*β*_5_	
≠0	0	0	GDP and carbon emissions show a linear relationship
<0	>0	0	GDP and carbon emissions show a U-shaped curve relationship
>0	<0	0	GDP and carbon emissions show an inverted U-shaped curve relationship
>0	<0	>0	GDP and carbon emissions show an N-curve relationship
<0	>0	<0	GDP and carbon emissions show an inverted N-curve relationship

**Table 2:** Descriptive statistical analysis of variables.

Variable	Meaning of Variables	Mean	Std. Dev.	Min	Max	Observations
*PCE*	ln*PCE*	1.6694	0.7830	−0.7729	3.6812	2160
*CTDR*	Whether to reform	0.1551	0.3621	0	1	2160
*GDP*	ln*GDP*	14.0540	1.0765	10.0858	16.7800	2160
*SI*	ln*SI*	102.432	16.164	60.383	170.451	2160
*GPE*	ln*GPE*	0.487	0.103	0.027	0.859	2160
*PD*	ln*PD*	6.1773	0.5597	4.3450	7.2858	2160
*CEI*	ln*CEI*	0.9324	0.5002	−1.6252	2.7398	2160

**Table 3:** Baseline regression results of the reform.

	*PCE*			
	(1)	(2)	(3)	(4)
Reform × After	0.875 ***	0.0340 **	0.2070 ***	0.0163 ***
	(20.56)	(2.09)	(9.73)	(2.72)
ln*PD*			–0.0669 ***	−0.3238 ***
			(−3.82)	(−20.28)
ln*SI*			0.7771 ***	0.0453 ***
			(21.33)	(5.45)
ln*CEI*			0.5640 ***	1.0963 ***
			(35.04)	(100.91)
ln*GPE*			0.2626 ***	−0.0445 ***
			(16.86)	(−6.27)
ln*GDP*			−4.0303 ***	−0.7188 ***
			(−2.99)	(−2.81)
ln*GDP*^2^			0.2424 **	0.1357 ***
			(2.47)	(7.27)
ln*GDP*^3^			−0.0054 **	−0.0035 ***
			(−2.28)	(−7.72)
Time fixed effects	No	Yes	No	Yes
Individual fixed effects	No	Yes	No	Yes
Observations	2160	2160	2160	2160
Adjust-R^2^	0.1634	0.9773	0.8244	0.9971
F	422.72	678.79	1268..34	5104.04

*t* values are in parentheses, *** *p* < 0.01, ** *p* < 0.05.

**Table 4:** Regional heterogeneity effects of reform.

	*PCE*			
	(1) Center	(2) Periphery	(3) Center	(4) Periphery
Reform × After	−0.0566 *	0.1220 ***	−0.0427 ***	0.0656 ***
	(−1.87)	(6.36)	(−3.63)	(8.63)
ln*PD*			−0.3855 ***	−0.2729 ***
			(−9.28)	(−15.86)
ln*SI*			−0.0022	0.0414 ***
			(−0.07)	(5.13)
ln*CEI*			1.1841 ***	1.0450 ***
			(41.40)	(89.27)
ln*GPE*			−0.0321 **	−0.0473 ***
			(−2.10)	(−5.96)
ln*GDP*			4.6353 ***	−0.9179 ***
			(2.72)	(−3.69)
ln*GDP*^2^			−0.2234 *	0.1510 ***
			(−1.88)	(8.26)
ln*GDP*^3^			0.0047 *	−0.0038 ***
			(1.70)	(−8.69)
Constant term	0.8573 ***	0.8586 ***	−31.0090 ***	−3.4104 ***
	(21.21)	(30.62)	(−3.87)	(−3.00)
Double fixed effects	Yes	Yes	Yes	Yes
Observations	432	1728	432	1728
Adjust-R^2^	0.9674	0.9797	0.9958	0.9974
F	313	738.90	2152.99	5584.03

*t* values are in parentheses, *** *p* < 0.01, ** *p* < 0.05, * *p* < 0.1.

**Table 5:** Placebo test of reform.

	*PCE*	
	(1)	(2)
5th year before reform	−0.0124	
	(−1.04)	
4th year before reform	−0.0030	
	(−0.25)	
3rd year before reform	−0.0071	
	(−0.62)	
2nd year before reform	0.0020	
	(0.20)	
Year 1 before reform	0.0072	
	(0.77)	
The year of reform	0.0055	−0.0244
	(0.67)	(−1.08)
The 1st year after reform		−0.0038
		(−0.17)
The 2nd year after reform		0.0347
		(1.45)
The 3rd year after reform		0.0374
		(1.50)
The 4th year after reform		0.0592 **
		(2.38)
The 5th year after reform		0.0514 **
		(1.99)
Time fixed effects	Yes	Yes
Individual fixed effects	Yes	Yes
Observations	2160	2160
Adjust-R^2^	0.9971	0.9773
F	4909.14	656.38

*t* values are in parentheses, ** *p* < 0.05.

**Table 6:** Robustness tests.

	*PCE*			
	2000–2003		2009–2017	
	(1) Center	(2) Periphery	(3) Center	(4) Periphery
Reform × After	0.0331 ***	0.0977 ***	0.0174 ***	0.0308 ***
	(5.15)	(11.56)	(4.27)	(6.26)
Control variables	Yes	Yes	Yes	Yes
Time fixed effects	Yes	Yes	Yes	Yes
Individual fixed effects	Yes	Yes	Yes	Yes
Observations	480	384	1080	864
Adjust-R^2^	0.9995	0.9995	0.9991	0.9992
F	7303.2	7634	9195.74	10,319.30

*t* values are in parentheses, *** *p* < 0.01.

**Table 7:** Mechanism test.

	Explained Variables		
	*PD*	*CEI*	*SI/TI*
Overall area	0.0130	−0.0724 ***	−0.1433 ***
	(1.56)	(−3.64)	(−3.94)
Central Cities	0.0667 ***	−0.1763 ***	−0.0458
	(4.18)	(−4.28)	(−0.74)
Peripheral Cities	−0.0326 ***	0.0557 ***	−0.1976 ***
	(−3.26)	(2.65)	(−4.26)

*t* values are in parentheses, *** *p* < 0.01.

## Data Availability

Not applicable.
